# Light Microclimate-Driven Changes at Transcriptional Level in Photosynthetic Grape Berry Tissues

**DOI:** 10.3390/plants10091769

**Published:** 2021-08-25

**Authors:** Andreia Garrido, Ric C. H. De Vos, Artur Conde, Ana Cunha

**Affiliations:** 1 Centre of Molecular and Environmental Biology (CBMA), Department of Biology, University of Minho, Campus de Gualtar, 4710-057 Braga, Portugal; arturconde@bio.uminho.pt; 2 Centre for the Research and Technology of Agro-Environmental and Biological Sciences (CITAB), University of Trás-os-Montes e Alto Douro, Quinta de Prados, 5000-801 Vila Real, Portugal; 3 Business Unit Bioscience, Wageningen Plant Research, Wageningen University and Research (Wageningen-UR), P.O. Box 16, 6700 AA Wageningen, The Netherlands; ric.devos@wur.nl; 4 Centre of Biological Engineering (CEB), University of Minho, Campus de Gualtar, 4710-057 Braga, Portugal

**Keywords:** light microclimate, exocarp, seed, gene expression, enzyme activity, grape berry photosynthesis, metabolic pathways

## Abstract

Viticulture practices that change the light distribution in the grapevine canopy can interfere with several physiological mechanisms, such as grape berry photosynthesis and other metabolic pathways, and consequently impact the berry biochemical composition, which is key to the final wine quality. We previously showed that the photosynthetic activity of exocarp and seed tissues from a white cultivar (Alvarinho) was in fact responsive to the light microclimate in the canopy (low and high light, LL and HL, respectively), and that these different light microclimates also led to distinct metabolite profiles, suggesting a berry tissue-specific interlink between photosynthesis and metabolism. In the present work, we analyzed the transcript levels of key genes in exocarps and seed integuments of berries from the same cultivar collected from HL and LL microclimates at three developmental stages, using real-time qPCR. In exocarp, the expression levels of genes involved in carbohydrate metabolism (*VvSuSy1*), phenylpropanoid (*VvPAL1*), stilbenoid (*VvSTS1*), and flavan-3-ol synthesis (*VvDFR*, *VvLAR2*, and *VvANR*) were highest at the green stage. In seeds, the expression of several genes associated with both phenylpropanoid (*VvCHS1* and *VvCHS3*) and flavan-3-ol synthesis (*VvDFR* and *VvLAR2*) showed a peak at the *véraison* stage, whereas that of RuBisCO was maintained up to the mature stage. Overall, the HL microclimate, compared to that of LL, resulted in a higher expression of genes encoding elements associated with both photosynthesis (*VvChlSyn* and *VvRuBisCO*), carbohydrate metabolism (*VvSPS1*), and photoprotection (carotenoid pathways genes) in both tissues. HL also induced the expression of the *VvFLS1* gene, which was translated into a higher activity of the FLS enzyme producing flavonol-type flavonoids, whereas the expression of several other flavonoid pathway genes (e.g., *VvCHS3*, *VvSTS1*, *VvDFR*, and *VvLDOX*) was reduced, suggesting a specific role of flavonols in photoprotection of berries growing in the HL microclimate. This work suggests a possible link at the transcriptional level between berry photosynthesis and pathways of primary and secondary metabolism, and provides relevant information for improving the management of the light microenvironment at canopy level of the grapes.

## 1. Introduction

Grapevine (*Vitis vinifera* L.) is commonly cultivated across temperate to semi-dry areas, including the Mediterranean region [1,2]. Currently, grape berry and wine production are affected by the escalation of environmental constraints, due to the intensification of climate change. Thus, adaptation and/or stress mitigation strategies are being implemented for a better management of vineyards (as reviewed by Santos et al. [3]).

Grape berry is composed of different tissues and cell layers, including the exocarp (skin), mesocarp (pulp), and seeds, which present distinct anatomical characteristics and biochemical profiles during development [4,5,6]. Different tissues of the grape berry have different functions, mainly anatomical/structural, physiological, and ecological, but they are also important in viniculture because their composition has a direct impact on the wine organoleptic properties (e.g., color, aroma, flavor, and texture) [7,8]. The exocarp contributes to the integrity of the whole berry by protecting the inner tissues from mechanical damage or pathogen attack [6], allowing timely seed dissemination [9], and also confers protection against ultraviolet light exposure, especially due to its flavonols content [10]. The seed is rich in flavan-3-ol monomers and procyanidins (tannins), which confer protection against herbivory but are also responsible for the bitterness and astringency of the wine [11]. Both primary and secondary metabolites of grape berry tissues are extremely important for fruit nutritional and organoleptic characteristics [12]. Complex regulatory mechanisms are involved in their synthesis, such as many transcriptional, translational and biochemical mechanisms, which can be also modulated by biotic and abiotic factors (as reviewed by Serrano et al. [13]).

It is well established that environmental conditions have a strong influence on metabolism of grape berry cells [14]. Light is an abiotic factor that influences the overall grapevine physiology and grape berry composition [15]. Thus, viticulture practices that involve canopy manipulations (e.g., leaf removal, shading covering, canopy conduction systems, and irrigation) are directly related to the levels of light reaching the leaves and grape berry clusters [16]. Smart et al. [17] introduced the concept of the microclimate to define the specific environmental conditions in the vicinity of leaves and fruits. The quality of light regimes experienced by grape berries, either the visible light or the ultraviolet radiation [18,19], has effects on their primary metabolites, such as amino acids, sugars and organic acids [20,21], and chlorophylls and carotenoids [22], in addition to their secondary metabolites, such as phenylpropanoids, flavonoids, and flavonols [10,18,19,23,24]. In addition, some studies also addressed these effects at the transcriptional level [18,19,22,23,25]. However, although this direct or indirect regulation by light is well understood in a number of pathways (for example, for flavonoid and anthocyanin pathways [26], and chlorophyll and carotenoid pathways [22,23]) for other pathways, this is not the case. Moreover, most of these studies focused on whole berries or just the skin, rather than other tissues/organs such as seeds. To the best of our knowledge, no studies concerning the effects of the light microclimate on grape seed metabolism and gene expression have been reported to date.

Like leaves, fruits may present photosynthetic activity, at least at their early stage of development [27,28]. In grape berries, both the exocarp and the seed outer integument exhibit photosynthetic activity [29] and their photosynthetic competence is responsive to the light microclimate experienced by the grapes throughout their development [30]. More recently, we characterized the photosynthetic profiles of these two berry tissues collected from clusters growing in two contrasting light microclimates in the canopy: LL (low light), i.e., shaded inner zones of the canopy; and HL (high light), i.e., grape berry clusters are exposed to direct sunlight part of the day, receiving three-fold more light intensity than LL clusters [31]. Moreover, the photosynthetic profiles of these two berry tissues were also assessed in vineyards under short-term mitigation treatments against climate adversities, i.e., foliar kaolin application and soil irrigation [31]. A metabolomics study showed that both light microclimate and irrigation were the main environmental factors influencing the metabolite composition of exocarp and seed [32].

Transcriptomics and genomics studies have disclosed the main elements involved in berry photosynthesis, especially in the skin [33,34,35,36,37]. In particular, Waters et al. [37], using cDNA microarray analysis, verified that expressed sequence tags (ESTs) involved in photosynthesis and carbohydrate metabolism were co-regulated, suggesting that photosynthesis in the berry skin is a source of carbohydrate for the berry skin itself. However, much uncertainty remains about the relationship between grape berry photosynthesis and its primary and secondary metabolism, and whether this is regulated at the level of gene transcription or enzyme activity.

The main objective of the present work was to evaluate the transcriptional changes in key genes involved in photosynthesis, sucrose metabolism, and secondary metabolite pathways (carotenoids, phenolics), in both the exocarp and seed of white grape berries exposed to either the HL or LL microclimate, aiming to establish a potential link between transcripts, metabolites [32], and photosynthetic activity [31] in these berry tissues.

## 2. Results and Discussion

### 2.1. Transcriptional Changes in Photosynthetic Machinery Elements and Primary Metabolism

**Figure 1:**
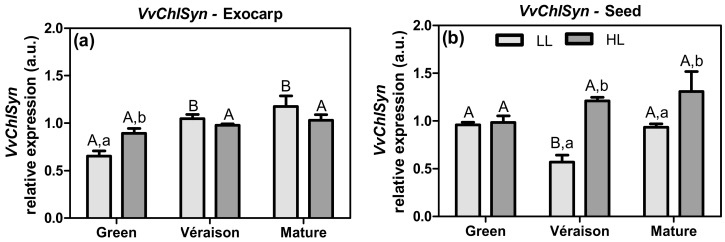
Relative expression in arbitrary units (a.u.) of transcripts of chlorophyll synthase gene (*VvChlSyn*) in (**a**) exocarp and (**b**) seeds, from berries grown at two distinct light microclimates (LL and HL) and at three developmental stages (green, *véraison*, and mature), as determined by real-time qPCR. Expression levels are normalized to the mean expression of reference genes *VvACT1* and *VvGAPDH*. Statistical analysis (two-way ANOVA, *p* ≤ 0.05, *n* = 3) was applied after data Log(X+1) transformation. Statistical notation: capital letters refer to differences between developmental stages for the same microclimate, and lowercase letters refer to differences between microclimates for each stage. When the letters are omitted, it means that the respective factor did not have a significant effect.

Chlorophylls are crucial elements from thylakoid membranes in the chloroplast, and are responsible for light energy harvesting, together with the carotenoids, and for initiating the flow of electrons into the electron transport chain. We analyzed the transcription of the *Chlorophyll Synthase* gene (*VvChlSyn*) in exocarp and seed (Figure 1). *VvChlSyn* codes for chlorophyll synthase (Enzyme Commission Number—EC 2.5.1.62), the enzyme that catalyzes the last step of the biosynthetic pathway of chlorophylls *a* and *b*.

Regarding the microclimate effects on *VvChlSyn* expression in the exocarp, statistical differences between HL and LL were observed already at the green stage, in which HL led to a higher expression compared with LL (Figure 1a). During berry development, this *VvChlSyn* expression remained stable in HL exocarps, whereas it significantly increased (1.8-fold) in LL exocarps (Figure 1a). These results for *VvChlSyn* expression in the exocarp are in agreement with the chlorophyll levels previously reported in this tissue [31], and are also consistent with the observation that HL exocarps from green berries present a higher photosynthetic activity than LL exocarps [31]. In seeds, both light microclimates showed similar *VvChlSyn* expression at the green stage, whereas at both later stages (*véraison* and mature) HL significantly increased *VvChlSyn* expression (2.1 and 1.4-fold at *véraison* and mature stages, respectively), compared to LL (Figure 1b). The inner localization of seeds causes constrictions in the level of light received, especially at the later stages of development, when the volume of the fruit increases [27]. Thus, this result of *VvChlSyn* expression in seeds may be partly explained by the dependence of these inner tissues on high light intensities, and consistent with HL seeds being able to acclimate the photosynthetic capacity to higher light intensity challenges [30]. Overall, these results highlight the importance of the HL microclimate on the *VvChlSyn* expression, which can also be reflected in the chlorophyll levels and in a functional photosynthetic system of both grape berry tissues.

Carotenoids not only contribute to the color and, through their cleavage into aromatic apocarotenoids such as ionones, to the aroma of fruits, but are also important components of the photosynthetic apparatus, by playing a key role in light harvesting and transference of energy in the photosystems [38]. The carotenoid biosynthetic pathway comprises the synthesis of carotenes and their subsequent conversion into xanthophylls [39]. In land plants, the thermal dissipation of the excess light energy is performed by two xanthophyll cycles [39]: (1) the violaxanthin (Vx) cycle, i.e., the reversible enzymatic conversion of violaxanthin to zeaxanthin, via the intermediate antheraxanthin; and (2) the lutein-epoxide (Lx) cycle, i.e., the reversible enzymatic conversion of Lx to lutein.

**Figure 2:**
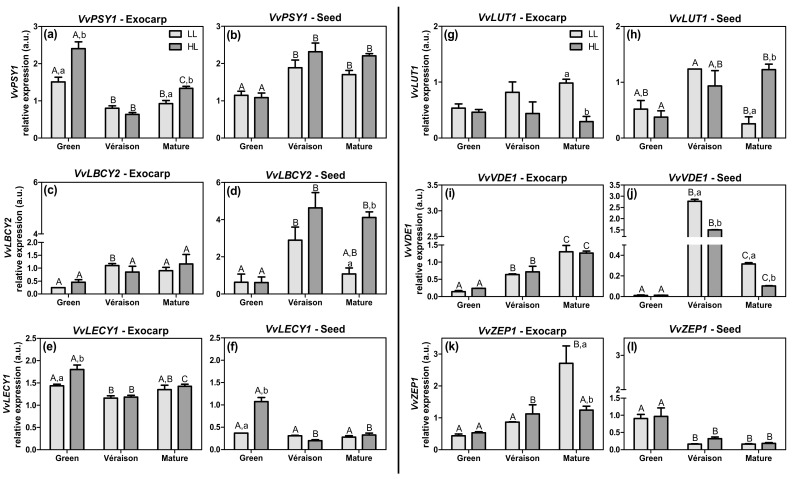
Relative expression in arbitrary units (a.u.) of transcripts of phytoene synthase 1 (*VvPSY1*) (**a**,**b**), lycopene beta-cyclase 2 (*VvLBCY2*) (**c**,**d**), lycopene epsilon cyclase 1 (*VvLECY1*) (**e**,**f**), carotene epsilon-monooxygenase or lutein-deficient 1 (*VvLUT1*) (**g**,**h**), violaxanthin de-epoxidase 1 (*VvVDE1*) (**i**,**j**), and zeaxanthin epoxidase 1 (*VvZEP1*) gene (**k**,**l**) in exocarp and seeds, from berries grown at two distinct light microclimates (LL and HL), and at three developmental stages (green, *véraison*, and mature), as determined by real-time qPCR. Expression levels are normalized to the mean expression of reference genes *VvACT1* and *VvGAPDH*. Statistical analysis (two-way ANOVA, *p* ≤ 0.05, *n* = 3) was applied after data Log(X+1) transformation. Statistical notation: capital letters refer to differences between developmental stages for the same microclimate, and lowercase letters refer to differences between microclimates for each stage. When the letters are omitted, it means that the respective factor did not have a significant effect.

In the present work, we analyzed the expression of several genes coding for key enzymes of the carotenoid pathway (Figure S1), namely: *phytoene synthase 1* (*VvPSY1*) (Figure 2a,b), which encodes the first dedicated carotenoid biosynthetic enzyme (EC 2.5.1.32); *lycopene beta-cyclase 2* (*VvLBCY2*) (Figure 2c,d), a critical gene diverting to the branch of β-carotene and the violaxanthin cycle, and thus competing with *lycopene epsilon cyclase 1* (*VvLECY1*) (Figure 2e,f). The latter is involved in the conversion of lycopene into δ-carotene; that is, in the direction of α-carotene and lutein synthesis, diverting to the lutein-epoxide cycle; *carotene epsilon-monooxygenase* or *lutein-deficient 1* (*VvLUT1*) (Figure 2g,h), which is responsible for the synthesis of lutein and the lutein-epoxide cycle [39]; *violaxanthin de-epoxidase 1* (*VvVDE1*) and *zeaxanthin epoxidase 1* (*VvZEP1*) (Figure 2i–l), which are both involved in the violaxanthin cycle.

The expression pattern of all genes was altered during berry development (Figure 2), suggesting the importance of the ripening-dependent changes in carotenoid composition in both photosynthetic tissues. The results for both *VvPSY1*, *VvLBCY2*, and *VvLECY1* (Figure 2a–f) show that, at specific points of berry development, the HL berries exhibited a significantly increased expression in either exocarp or seed, in comparison to LL berries. This result suggests a higher carotenoid biosynthesis in fully exposed berries compared to shaded berries. Although *VvPSY1* expression peaked at the green stage in the exocarp (Figure 2a), both *VvPSY1* and *VvLBCY2* expressions tended to increase during berry development in both tissues, most clearly in HL seeds (Figure 2a–d). In contrast, *VvLECY1* expression decreased from green to mature, again especially in HL seeds (Figure 2e,f), suggesting an up-regulation of the Vx route instead of Lx at later stages. This higher expression of *VvLECY1* in both tissues of green HL berries, together with relatively low levels of *VvLBCY2* (Figure 2c,d), point to an up-regulation of the Lx route at the green stage. In exocarps from the green stage, the higher expression of genes *VvPSY1* and *VvLECY1* in HL (Figure 2a,e) is in accordance with the higher total carotenoid content (i.e., considering the summed values of α- and β-carotene and lutein) in these HL berries compared to LL berries [31]. Moreover, the reduction of the expression of these two genes in the exocarp throughout berry development (Figure 2a,e) is also in line with the previously reported decrease in total carotenoid levels [31]. These results suggest that, during their early development, the green berries growing in the HL microclimate, i.e., with their exocarp exposed to direct sunlight, acclimate to the relative high light levels via an increased carotenoid/xanthophyll biosynthesis, supporting their higher photosynthetic activity [31]. Similarly, previous studies using berries from a different white grape variety (Sauvignon Blanc) report that, at their green stage, the exposed berries contained a higher pool of carotenoids compared to shaded berries, and were more acclimated to light stress [23,40].

The transcript levels of genes directly involved in the xanthophyll cycles (*VvLUT1*, *VvVDE1*, and *VvZEP1*—Figure 2g–l) revealed that: in the exocarp of mature grapes, *VvLUT1* was significantly (>3-fold) less expressed in the HL microclimate compared to the LL microclimate (Figure 2g), whereas in seeds of the same stage the opposite result was found (Figure 2h); the *VvVDE1* expression in the exocarp increased throughout berry development and was similar between HL and LL conditions (Figure 2i), whereas in seeds its expression was lower in HL than in LL, except for the green stage, and clearly peaking at the *véraison* stage (Figure 2j); *VvZEP1* expression in the exocarp increased during development, reaching significantly higher levels (>2-fold) at the mature stage in LL berries than in HL berries (Figure 2k); in contrast, its expression in seeds decreased equally in the HL and LL berries (Figure 2l). The increase in *VvVDE1* expression throughout exocarp development (Figure 2i) suggests that the Vx cycle is up-regulated in this external tissue, thereby protecting the photosynthetic apparatus from damage during berry development and ripening. Indeed, in the Sauvignon Blanc variety, both *VvVDE1* expression and the pool of carotenoids (predominantly xanthophylls) increased in light-exposed berries, compared to shaded ones, and were able to protect the photosynthetic machinery [22]. 

In the mature seed, the maintenance of the higher expressions in HL compared to LL of both *VvLUT1* (Figure 2h) and *VvLBCY2* (Figure 2d), together with the stronger decrease in *VvVDE1* expression (Figure 2j), are indicative of an up-regulation of both lutein and zeaxanthin synthesis by HL. In addition, in seeds the increase in *VvVDE1* expression and decrease in *VvZEP1* from green to *véraison* stages (Figure 2j,l) suggests the promotion of the zeaxanthin biosynthetic pathway in this tissue, upon berry development. 

Globally, at the green stage, the higher expression of *VvLECY1* in both tissues (Figure 2e,f), together with the reduced levels of *VvLBCY2* (Figure 2c,d) and *VvLUT1* (Figure 2g,h), suggest that the carotenoid pathway branches towards α-carotene, which seems to play a role in both tissues at this initial stage of development. At *véraison* and mature stages, the exocarp relies on Vx, reducing the carbon flow to lutein synthesis (Figure 2e,g,i,k), whereas in seeds both xanthophyll cycles appear to operate at these later stages of development (Figure 2d,h,j). 

**Figure 3:**
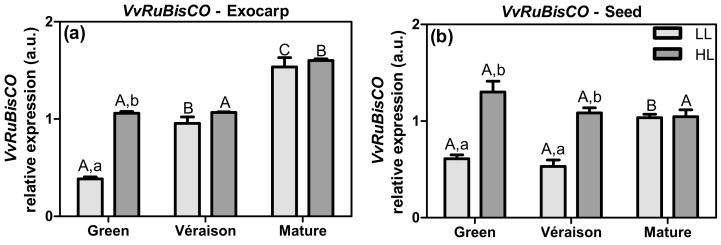
Relative expression in arbitrary units (a.u.) of transcripts of ribulose bisphosphate carboxylase/oxygenase gene (*VvRuBisCO*) in (**a**) exocarp and (**b**) seeds, from berries grown at two distinct light microclimates (LL and HL), and at three developmental stages (green, *véraison*, and mature), as determined by real-time qPCR. Expression levels are normalized to the mean expression of reference genes *VvACT1* and *VvGAPDH*. Statistical analysis (two-way ANOVA, *p* ≤ 0.05, *n* = 3) was applied after data Log(X+1) transformation. Statistical notation: capital letters refer to differences between developmental stages for the same microclimate, and lowercase letters refer to differences between microclimates for each stage. When the letters are omitted, it means that the respective factor did not have a significant effect.

Ribulose bisphosphate carboxylase/oxygenase (RuBisCO; EC 4.1.1.39) is a critical enzyme of the Calvin–Benson cycle of photosynthesis that catalyzes the condensation of CO_2_ with ribulose 1,5-bisphosphate (RuBP), producing two molecules of 3-phosphoglycerate (3-PGA) as the stable products. To determine the effect of the grape berry microclimate on this key enzyme in photosynthesis, the relative expression of the *VvRuBisCO* gene in grape berry exocarps and seeds was analyzed in both microclimates (Figure 3). 

At the green stage, both exocarps and seeds from HL berries showed a significantly higher expression of *VvRuBisCO* (>2-fold), in comparison to LL berries (Figure 3a,b), in parallel with our previous results regarding their photosynthetic activities [31]. At the *véraison* stage, the HL microclimate led to a higher *VvRuBisCO* expression in seeds only (Figure 3b). No difference between HL and LL was observed at the mature stage in either tissue. These results suggest that the microclimate had an impact on carbon fixation capacity of both tissues, most specifically in immature fruits. 

During berry development, the relative expression of *VvRuBisCO* in HL seeds was more or less constant, whereas for exocarps it increased in both microclimates (Figure 3). These results are in accordance with the photosynthetic activity pattern of exocarps, which was maintained at a high level until later ripening stages, whereas in seeds this activity declined *post-véraison* [30,31]. During grape berry development, stomatal conductance decreases and gas exchanges with the atmosphere fall to virtually nil after *véraison* due to blockage of the stomata with cuticular waxes [41], so the maintenance/increase in *VvRuBisCO* expression during ripening may be related to the fixation of internally produced CO_2_. It is argued that refixation of respiratory CO_2_ by RuBisCO after the onset of ripening (e.g., from malate catabolism) can provide carbon skeletons for other metabolic pathways, including seed storage lipids, as proposed for oilseed rape [42,43]. 

**Figure 4:**
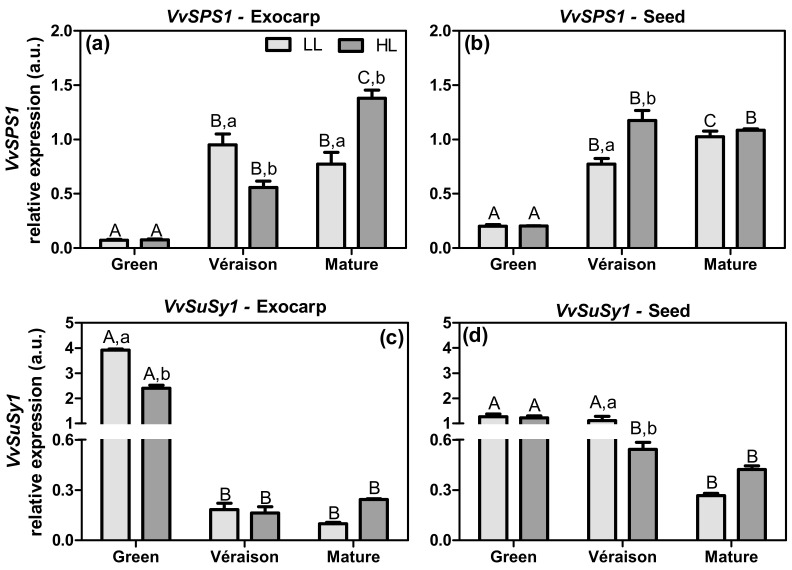
Relative expression in arbitrary units (a.u.) of transcripts of sucrose-phosphate synthase 1 (*VvSPS1*) (**a**,**b**) and sucrose synthase (*VvSuSy1*) gene (**c**,**d**) in exocarp and seeds, from berries grown at two distinct light microclimates (LL and HL), and at three developmental stages (green, *véraison*, and mature), as determined by real-time qPCR. Expression levels are normalized to the mean expression of reference genes *VvACT1* and *VvGAPDH*. Statistical analysis (two-way ANOVA, *p* ≤ 0.05, *n* = 3) was applied after data Log(X+1) transformation. Statistical notation: capital letters refer to differences between developmental stages for the same microclimate, and lowercase letters refer to differences between microclimates for each stage. When the letters are omitted, it means that the respective factor did not have a significant effect.

Carbohydrate metabolism is a key point of interconnection between several metabolomic pathways, contributing to the synthesis of intermediate compounds [12,44]. In the present work, we analyzed the expression of two genes *VvSPS1* and *VvSuSy1* (Figure 4), both coding important enzymes in sugar metabolism. 

Sucrose-phosphate synthase (SPS; EC 2.4.1.14) is an enzyme involved in the sucrose biosynthesis, catalyzing the formation of sucrose-6-phosphate from uridine diphosphate glucose (UDP-glucose) and fructose-6-phosphate [34]. In both tissues, the relative transcript levels of *VvSPS1* increased from green to later stages of berry development, being responsive to light microclimate (Figure 4a,b). The main difference between these tissues is that, in the exocarp, the HL microclimate, compared to LL, led to a significant up-regulation of *VvSPS1* expression at the mature stage, whereas in seeds this effect was observed at the earlier *véraison* stage. These results reveal that sucrose biosynthesis was enhanced in exocarps and seeds during berry ripening. Previously, we showed that the exocarp tissue is photosynthetically active until the mature stage of berry ripening, whereas the seeds show a slight decrease in photosynthesis, especially after the *véraison* stage [31]. The expression patterns of *VvSPS1* (Figure 4) and *VvRuBisCO* (Figure 3) were quite similar, suggesting an active photosynthetic/Calvin–Benson cycle function until late in berry development in both grape berry tissues, and particularly in the exocarp. In this manner, from the *véraison* stage onwards, a flux of triose phosphates from berry chloroplasts can fuel the biosynthesis of other intermediates and products, as the cytosolic synthesis of sucrose, the major translocatable product of photosynthesis. Supporting this suggestion, Wu et al. [45] showed that the enzyme activity of SPS in whole grape berries from several cultivars increased with development.

Sucrose synthase (SuSy; EC 2.4.1.13) is a glycosyl transferase, mainly present in the cytosol, that is responsible for both synthesis and catabolism of sucrose, and thus catalyzes the reversible conversion of sucrose and uridine diphosphate into UDP-glucose and fructose [46,47]. Wang et al. [48], suggested that in grape berries the SuSy might cooperate with cell wall invertases, cleaving the sucrose unloaded into the cytoplasm of sink cells. The products of sucrose cleavage by SuSy are available for many metabolic pathways, such as energy and primary metabolite production, and synthesis of complex carbohydrates (reviewed by Stein and Granot [47]). The grape genome contains five *SuSy* genes [46]. Both the exocarp and seed had high levels of *VvSuSy1* expression at the green stage, which decreased at later ripening stages, particularly in exocarp (Figure 4c,d), thus in the opposite pattern to that of *VvSPS1*. This relatively high expression level of *VvSuSy1* and low expression of *VvSPS1* at the green stage in both tissues may suggest that sucrose imported from leaves, rather than sucrose produced locally in the berry, is crucial to coping with the high carbon and energy demands associated with the intense cell proliferation activity that occurs during this phase of fruit growth [49].

### 2.2. Transcriptional and Biochemical Activity Changes in the Phenylpropanoid and Flavonoid Pathways

Both the expression patterns of *VvRuBisCO* and *VvSPS1*, and our previous metabolomics study [32], suggest a role of berry photosynthesis in metabolism. Possibly, this berry photosynthesis can contribute locally with energy and carbohydrate resources to cope with the high demand of substrates needed for secondary metabolism, as also noted by others [50,51,52,53,54]. We therefore also intended to analyze the effects of the two contrasting canopy light microclimates on the expression of genes involved in the phenylpropanoid, flavonoid, and stilbenoid pathways (Figure S2), which are responsible for the biosynthesis of key quality metabolites in both the exocarp and seed of the grape berry [55]. According to our previous metabolomics study [32], stilbenes were relatively abundant in both the green exocarp and mature seed, whereas flavonols were mostly detectable in the exocarp, especially at the *véraison* and mature stages, and flavan-3-ols were most abundant at the initial stages of development (i.e., green and *véraison*) in both the exocarp and seed. 

**Figure 5:**
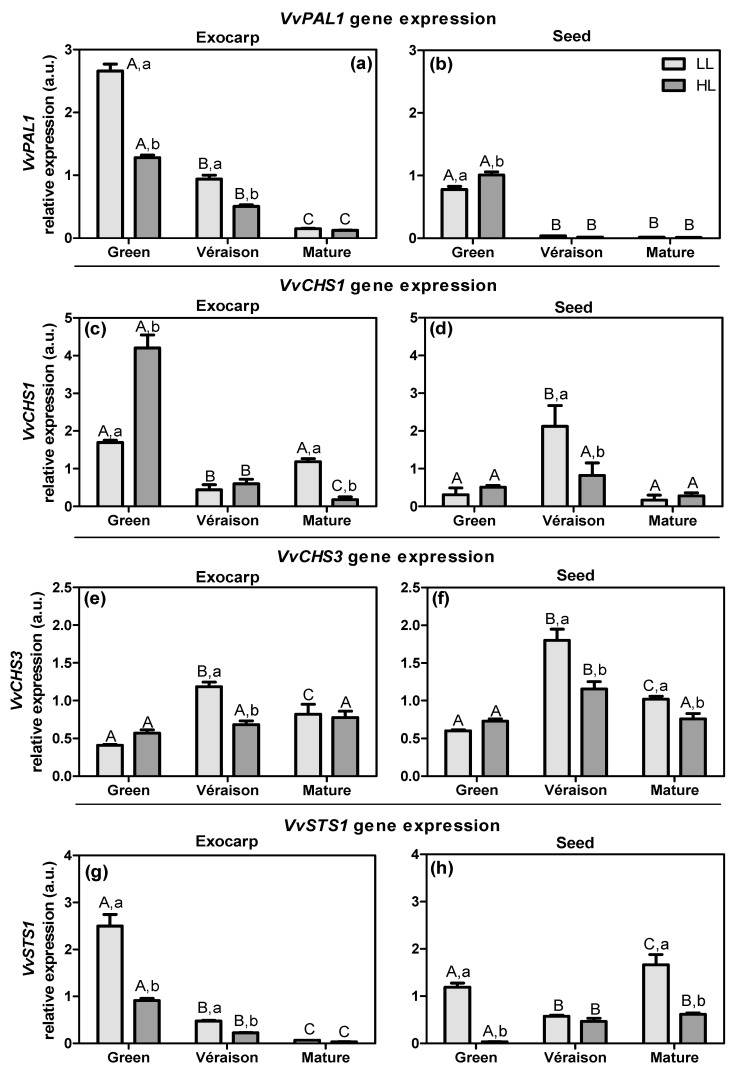
Relative expression in arbitrary units (a.u.) of transcripts of phenylalanine ammonia lyase 1 (*VvPAL1*) (**a**,**b**), chalcone synthase 1 and 3 (*VvCHS1* and *VvCHS3*) (**c**–**f**), and stilbene synthase 1 (*VvSTS1*) gene (**g**,**h**), in exocarp and seeds, from berries grown at two distinct light microclimates (LL and HL), and at three developmental stages (green, *véraison*, and mature), as determined by real-time qPCR. Expression levels are normalized to the mean expression of reference genes *VvACT1* and *VvGAPDH*. Statistical analysis (two-way ANOVA, *p* ≤ 0.05, *n* = 3) was applied after data Log(X+1) transformation. Statistical notation: capital letters refer to differences between developmental stages for the same microclimate, and lowercase letters refer to differences between microclimates for each stage. When the letters are omitted, it means that the respective factor did not have a significant effect.

The phenylpropanoid pathway starts with the conversion of phenylalanine into cinnamic acid by phenylalanine ammonia lyase enzyme (PAL; EC 4.3.1.24) [56]. In grapevine, the analysis of the genome predicts 13 copies for *PAL* genes [57], with *PAL1* the best characterized isoform [58]. In both berry tissues, the transcript levels of *VvPAL1* decreased with development (Figure 5a,b). The canopy microclimate effect was more evident for the exocarp, in which HL led to a decrease in *VvPAL1* expression compared to LL, at both green and *véraison* stages (Figure 5a). A previous study using berry skins of two red cultivars (Jingxiu and Jingyan) showed that *PAL* expression did not vary between exposed and shaded berries [59]. Another study with a red grape cultivar (Cabernet Sauvignon) showed that the expression levels of *PAL* in berry skin were up-regulated at both *véraison* and full mature stages upon increased sunlight exposure resulting from leaf removal [25]. These apparently conflicting results may be explained by the fact that these latter studies were focused on the later stages of development and involved red varieties, in which the anthocyanin biosynthesis and the expression of genes up- and downstream in the pathway, and their regulatory transcription factors (MYB family), are induced by light [60,61]. By comparison, the fact that *VvSuSy1* expression (Figure 4c) was higher in LL than HL at the green berry stage may have resulted in a higher content of hexoses, which are known to stimulate *PAL* expression [62,63], and thus may have been responsible for the higher expression of *PAL* in these LL exocarps (Figure 5a). Further studies are required to better understand and to confirm this hypothesis, including to determine the biochemical activity of both PAL and SuSy enzymes, and to study the expression of other already known isogenes. As noted above, the *PAL1* isoform is one of a total of 13 copies, but the enzyme activity may respond differently to the light microclimate and differ in the two grape berry tissues studied. However, previous studies showed that in the grape berries, grapevine leaves, and woody tissues, the expression levels of *VvPAL1* [64], *VvSPS1* [65], and *VvSuSy1* [66], respectively, were well correlated with the enzyme activities.

The flavonoid pathway is initiated by the chalcone synthase enzyme (CHS; EC 2.3.1.74) [67]. We analyzed the gene expression of two isoforms (i.e., *VvCHS1* and *VvCHS3*; Figure 5c–f), because in white grapevine varieties these are known to be mostly expressed within the estimated total of four isoforms [68]. In exocarp tissue, the expression of *VvCHS1* was highest at the green stage, particularly in HL berries, and decreased during subsequent ripening (Figure 5c). In seeds, *VvCHS1* was relatively low at the green stage and showed a peak at the *véraison* stage, particularly in LL berries (Figure 5d). Such a peak at the *véraison* stage with the highest levels in LL berries was also observed for the *VvCHS3* gene in both tissues (Figure 5d–f). Although *VvCHS3* exhibited a similar expression pattern in both tissues during development, the *VvCHS1* expression pattern appears to be dependent on the tissue and developmental stage, and was differentially regulated by the light microclimate. A previous study with grape berry from a red grapevine variety (Shiraz) showed that, for skin, and in contrast to our results, the expression of *VvCHS1* and *VvCHS3* increased during development, whereas for seeds, and in agreement with our results, the expression of both genes peaked at the *véraison* stage [69]. The increase in *VvCHS* expression in the skins of red varieties may be related to the ripening-induced biosynthesis of anthocyanins, which obviously does not take place in white cultivars. Moreover, cell suspension cultures of petiole *callus* from a white grapevine variety (Chardonnay) showed higher transcripts levels of *VvCHS1* when grown in the light compared to cells grown in the dark, whereas *VvCHS3* was not induced by light [69], in line with our results for exocarp from HL vs. LL at the green stage (Figure 5c,e).

Stilbene synthase enzyme (STS; EC 2.3.1.95) is responsible for the condensation of 4-coumaroyl-CoA with three molecules of malonyl-CoA producing resveratrol. In grapevine, the *VvSTS1* is the best characterized isoform from a total of 48 *STS* genes [70]. Its expression significantly decreased throughout exocarp development (Figure 5g) in a very similar pattern to that of *VvPAL1*. The HL microclimate led to a decrease in *VvSTS1* expression in the exocarp at the green stage only, whereas in seeds this decrease was observed at both green and mature stages (Figure 5g,h). Considering that CHS and STS compete for the same substrate (Figure S2), the opposite responses of the respective genes to the microclimate in the exocarp at the green stage (Figure 5c,g) may be indicative of a down-regulation of the stilbene branch by HL and a channeling of substrates into the flavonoid pathway at this early stage of the berry development. Clearly, further biochemical assays, such as the evaluation of STS and CHS activities, are needed to understand this opposite effect of HL on the stilbene and flavonoid branches. In the seeds, the expression of *VvSTS1* was particularly evident at the green and mature stages, in an inverse pattern compared to that of the *VvCHS1* and *VvCHS3* genes (Figure 5d,f,h).

**Figure 6:**
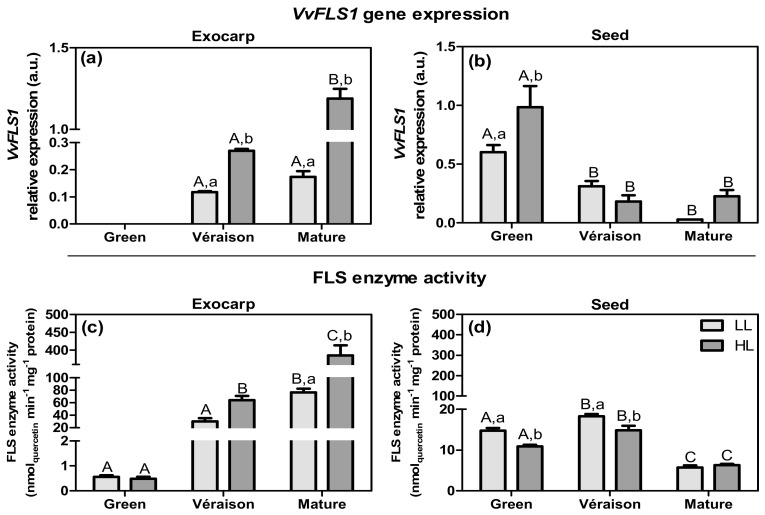
Relative expression in arbitrary units (a.u.) of transcripts of flavanol synthase 1 gene (*VvFLS1*) in (**a**) exocarp and (**b**) seeds, from berries grown at two distinct light microclimates (LL and HL) and at three developmental stages (green, *véraison*, and mature), as determined by real-time qPCR. Expression levels are normalized to the mean expression of reference genes *VvACT1* and *VvGAPDH*. Statistical analysis (two-way ANOVA, *p* ≤ 0.05, *n* = 3) was applied after data Log(X+1) transformation. Flavonol synthase (FLS) biochemical activity (expressed in V_max_ (nmol_quercetin_ min^−1^ mg^−1^ protein)) in (**c**) exocarp and (**d**) seeds, from berries grown at two distinct light microclimates (LL and HL) and at three developmental stages (green, *véraison*, and mature). Statistical analysis (two-way ANOVA, *p* ≤ 0.05, *n* = 5) was applied. Statistical notation: capital letters refer to differences between developmental stages for the same microclimate, and lowercase letters refer to differences between microclimates for each stage. When the letters are omitted, it means that the respective factor did not have a significant effect.

Flavonol synthase (FLS; EC 1.14.20.6) is the key enzyme for the biosynthesis of flavonol-type flavonoids, by catalyzing the oxidation of dihydroflavonols into flavonols. Among the five *FLS* genes in grape [71], only the isoform *FLS1* showed a clear expression pattern corresponding to the accumulation of flavonols in berry skins [72], and thus this gene was selected for our study. In the exocarp, the expression of *VvFLS1* was relatively low: it was undetectable at the green stage and increased subsequently (Figure 6a), in agreement with previous studies of both red and white grape berry skins [72]. In contrast, in seeds, *VvFLS1* expression was detected at all stages, and decreased as development progressed (Figure 6b). In both tissues, the HL microclimate, compared to LL, significantly up-regulated *VvFLS1* expression (e.g., a seven-fold increase for exocarp at the mature stage). To check if these changes in expression of the *FLS1* gene isoform were translated into changes in enzyme activity, we prepared enzyme extracts and determined the potential FLS activity (V_max_) using dihydroquercetin as a substrate. In the exocarp, the activity of the FLS enzyme showed a profile that was similar to that of the *VvFLS1* gene expression, both with respect to development (802-fold increase from green to mature) and microclimate (five-fold increase by HL at the mature stage) (Figure 6c). In seeds, FLS enzyme activity was significantly less different between green and *véraison* stages (1.2-fold), and decreased thereafter; in these first two stages, it was 1.3-fold higher under LL than in HL (Figure 6d). Thus, in contrast to exocarp, in seed this FLS enzyme activity only partly corresponded to the expression pattern of *VvFLS1* across sample groups.

In our previous metabolomics study, we showed that the HL microclimate led to an increase in total flavonols in the exocarp at all berry developmental stages [32]. Here, we showed that this HL microclimate also has a clear positive influence on both *VvFLS1* expression and total FLS enzyme activity in this tissue. This difference between microclimates can be explained by a light acclimation mechanism in the HL exocarps [23], thereby enhancing the biosynthesis of specific metabolites such as flavonols that can protect the photosynthetic apparatus from light-induced damage, in order to maintain exocarp photosynthesis until the end of berry development. It has frequently been suggested that flavonols can limit photodamage through their ability to scavenge ROS and other free radicals generated by photooxidation, thereby contributing to the maintenance of oxidative homeostasis [73]. Our results showing the up-regulation of *VvFLS1* expression by HL microclimate compared to LL are in accordance with previous studies performed with both white [23,74] and red [18,61] grape berry varieties, in addition to the observation that cell cultures from white berries exposed to high light levels showed an increase in *FLS* gene expression, compared to the cells in the dark condition [75]. 

Apart from light, other abiotic factors including temperature can also influence the flavonol branch of the flavonoid pathway. In the field, the effects of temperature and light received by the berry surface are not easily discernable. The LL and HL berry clusters had an average temperature of 26 °C and of 30 °C, respectively [31]. Recently, Gouot et al. [76] noted that, in general, high temperatures do not appear to directly affect the flavonol content, but can interfere negatively and indirectly through changes in gene expression and intermediates upstream of the flavonol branch and in the primary metabolism. Further research under controlled conditions is needed to better understand the overall grape berry biochemical responses to these combined abiotic factors.

The apparent discrepancy between *VvFLS1* gene expression and total FLS enzyme activity patterns observed in seeds may be due to other mechanisms associated with enzyme kinetics and/or post transcriptional regulation of this enzyme, and also due to the fact that FLS enzyme activity translates the sum of all five isoforms. In seeds, the abundance of flavonols was relatively low, as compared to exocarp, and not influenced by the berry microclimate [32]. In seeds from a red grape variety, the flavonols were even undetectable, and *VvFLS1* gene expression was very low and only detected at *post-véraison* stages [72]. Thus, in seeds, the influence of the microclimate on the flavonol branch of the flavonoid pathway is not as clear as in the exocarp. It is possible that, during seed maturation, other pathways, such as carotenoid biosynthesis, are more important as photooxidative protection mechanisms than the flavonol biosynthesis branch. In fact, at the green stage, *VvFLS1* expression was relatively high compared to that of both *véraison* and mature stages (Figure 6b), whereas the genes of the xanthophyll cycle were, in general, more expressed at the later stages of development (Figure 2).

**Figure 7:**
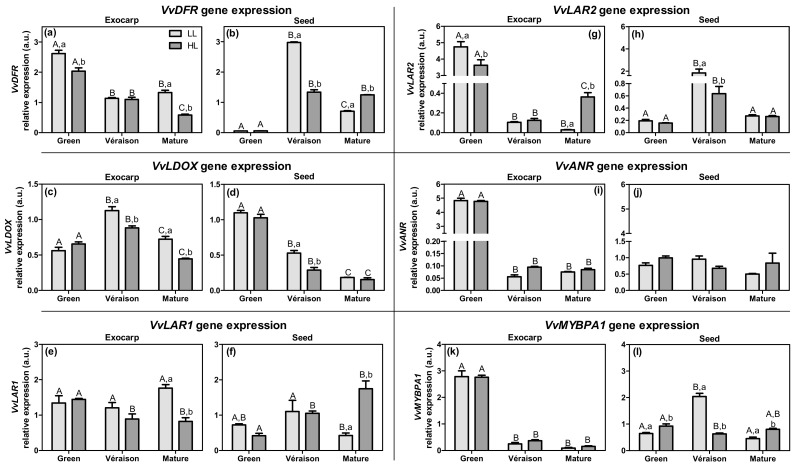
Relative expression in arbitrary units (a.u.) of transcripts of dihydroflavonol reductase (*VvDFR*) (**a**,**b**), leucoanthocyanidin dioxygenase (*VvLDOX*) (**c**,**d**), leucoanthocyanidin reductase 1 and 2 (*VvLAR1* and *VvLAR2*) (**e**–**h**), and anthocyanidin reductase (*VvANR*) gene (**i**,**j**), and of transcription factor MYBPA1 (*VvMYBPA1*) (**k**,**l**) in exocarp and seeds, from berries grown at two distinct light microclimates (LL and HL), and at three developmental stages (green, *véraison*, and mature), as determined by real-time qPCR. Expression levels are normalized to the mean expression of reference genes *VvACT1* and *VvGAPDH*. Statistical analysis (two-way ANOVA, *p* ≤ 0.05, *n* = 3) was applied after data Log(X+1) transformation. Statistical notation: capital letters refer to differences between developmental stages for the same microclimate, and lowercase letters refer to differences between microclimates for each stage. When the letters are omitted, it means that the respective factor did not have a significant effect.

Dihydroflavonols can also be converted into their corresponding leucoanthocyanidins, by the enzyme dihydroflavonol reductase (DFR; EC 1.1.1.219). These leucoanthocyanidins are subsequently converted into their corresponding anthocyanidins by the action of leucoanthocyanidin dioxygenase (LDOX; EC 1.14.20.4) (Figure S2). The leucoanthocyanidins and anthocyanidins are considered to be potential substrates for the formation of flavan-3-ols (also named flavanols or condensed tannins). Leucoanthocyanidin reductase (LAR; EC 1.17.1.3) converts leucocyanidin into catechin, whereas anthocyanidin reductase (ANR; EC 1.3.1.77) converts cyanidin into epicatechin. The transcript levels of genes related to flavan-3-ol biosynthesis are represented in Figure 7. 

In exocarp, *VvDFR* expression was highest in the green stage and decreased with ripening (Figure 7a). In contrast, seeds exhibited a residual expression level at the green stage, and a high level at *véraison*, which decreased again to the mature stage (Figure 7b). The expression of *VvDFR* was generally significantly higher in LL compared to HL (Figure 7a,b). The expression of leucoanthocyanidin dioxygenase (*VvLDOX*)—the route to the epicatechins—hardly changed in the exocarp during berry development among the genes tested (Figure 7c), whereas in seeds it decreased with ripening to relatively low levels (Figure 7d).

For the leucoanthocyanidin reductase—the route to the catechins—the expression of the genes *VvLAR1* and *VvLAR2*, coding for the only two isoforms identified in grapevine [57], were analyzed (Figure 7e–h). *VvLAR1* expression was roughly constant during ripening in both exocarp and seed. There was a significant difference between tissues in their microclimate responses at the mature stage: in the exocarp, the transcript levels were significantly lower (two-fold) in HL berries compared to LL berries (Figure 7e), whereas in seeds they were significantly higher in HL (4.2-fold) (Figure 7f). For both tissues, the transcript levels of *VvLAR2* varied more between ripening stages than the transcripts of *VvLAR1*, with two clear peaks—at the green stage for exocarp and at the *véraison* stage for seed (Figure 7g,h), which is in accordance with previous work with skins and seeds from a red grape variety [77]. Overall, the expression patterns of *VvLAR1* and *VvLAR2* appear to correspond to that of *VvDFR* (Figure 7a,b,e–h). Moreover, the Pearson correlation showed that both *VvDFR* and *VvLAR2* were significantly and positively correlated in both the exocarp (Table S2; *r* = 0.84, *p* < 0.0001) and seed (Table S3; *r* = 0.75, *p* < 0.001).

The relative expression level of *VvANR*, coding for anthocyanidin reductase, in the green exocarp was the highest of all the analyzed genes. However, this high expression in the exocarp was only observed at the green stage, and its transcript levels at subsequent developmental stages were negligible and markedly lower than the levels observed in seeds at all stages (Figure 7i,j). In seeds, *VvANR* transcript levels were maintained constant during ripening, but at relatively low levels (Figure 7j).

The transcription factor *VvMYBPA1* in the exocarp had a strong and significant positive correlation with genes related to flavan-3-ol biosynthesis (i.e., *VvDFR*, *VvLAR1*, *VvLAR2*, and *VvANR*), whereas in seed this transcription factor had a significant correlation with only two of these genes (*VvDFR* and *VvLAR2*) (Figure 7, and Tables S2 and S3). These results confirm its proposed role in regulating these structural flavonoid biosynthesis pathway genes [78,79].

**Figure 8:**
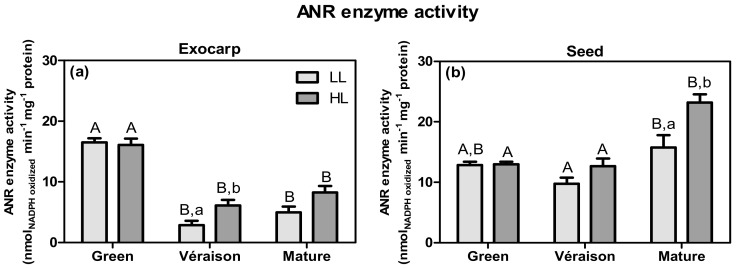
Anthocyanidin reductase (ANR; EC 1.3.1.77) biochemical activity, expressed in V_max_ (nmol_NADPH oxidized_ min^−1^ mg^−1^ protein) in exocarp (**a**) and seeds (**b**), from berries grown at two distinct light microclimates (LL and HL) and at three developmental stages (green, *véraison* and mature). Statistical analysis (two-way ANOVA, *p* ≤ 0.05, *n* = 4–8) was applied. Statistical notation: capital letters refer to differences between developmental stages for the same microclimate, and lowercase letters refer to differences between microclimates for each stage. When the letters are omitted, it means that the respective factor did not have a significant effect.

The activity of anthocyanidin reductase enzyme was also assessed to investigate whether the observed changes in the *VvANR* gene expression were translated at the enzyme activity level (Figure 8). In the exocarp, the ANR activity (V_max_) was highly consistent with the profile of its gene expression during development (compare Figure 8a with Figure 7i). For seeds, at the green and *véraison* stages, the ANR activity was also similar to its gene expression, but it significantly increased at the mature stage for both microclimates, especially in HL (Figure 8b). Although *VvANR* was the only gene whose expression was not significantly affected by the light microclimate (Figure 7i,j), the ANR enzyme activity was stimulated by HL at the *véraison* and mature stages, in the exocarp and seeds, respectively (Figure 8). This difference between ANR transcript levels and enzyme activity may be explained by other mechanisms associated with enzyme kinetics and/or post transcriptional regulation, possibly regulated by light [61]. Eventual phenological displacements between LL and HL grapes can also explain the differences in their ANR enzyme activity. Zha et al. [80] showed that a lower light condition, due to berry bagging, led to a delay of berry maturation, i.e., a lower content of soluble sugars and anthocyanins, and thus less color of the skin, although the effects of the temperature on the phenology of grapes were not assessed.

It is worth noting that the significant differences registered in the enzyme specific activities (of both FLS and ANR) were not due to any constitutive difference in the soluble protein content between sample groups (data not shown).

Overall, during exocarp ripening there was a symmetry between the profiles of the gene expression of flavonols (Figure 6) and those of flavan-3-ols (Figure 7). The direction to flavan-3-ol synthesis was more evident at the green stage, considering the transcript levels of *VvDFR*, *VvLAR2*, and *VvANR* (Figure 7a,g,i), and the activity of the ANR enzyme (Figure 8a). In contrast, the *VvFLS1* expression in this external berry tissue was negligible in the green stage, but increased throughout ripening (Figure 6a) and in an opposite fashion to that seen for *VvDFR* (Figure 7a). The Pearson correlation matrix for the exocarp samples (Table S2) also showed that *VvFLS1* was negatively correlated with all the genes downstream in the flavonoid pathway, being significantly negatively correlated with *VvDFR* (*r* = −0.77, *p* < 0.001), which may be explained by the fact that both genes code for enzymes that compete for the same substrate, i.e., dihydroflavonols. This shift from the green to the mature stage is in accordance with the abundance profiles of the flavanols and flavonols detected in this exocarp tissue [32]. Moreover, *VvANR* and both isoforms of *VvLAR* were significantly positively correlated in exocarp (Table S2), suggesting that both pathways of flavan-3-ol monomer synthesis are active. 

In the case of seeds, this shift between the expression of genes coding the enzymes of the flavonols and flavan-3-ol branches was not as evident as in the exocarp. *VvFLS1* expression was high at the green stage (Figure 6b), and decreased thereafter, whereas *VvDFR* and *VvLAR2* expressions peaked at the *véraison* stage (Figure 7b,h). Similar to the exocarp, in the seeds the *VvFLS1* expression was negatively correlated with some genes downstream in the pathway, i.e., *VvDFR*, *VvLAR1*, and *VvLAR2*, although this correlation was not statistically different (Table S3). Furthermore, and in contrast to the exocarp, in the seeds the *VvFLS1* expression was significantly positively correlated with the expression of two genes, i.e., *VvLDOX* and *VvANR* (Table S3), although this correlation was only observed at the green stage (c.f. Figure 6b and Figure 7d). These results corroborate the profile of flavan-3-ol monomers during seed development.

## 3. Materials and Methods

### 3.1. Grapevine Field Conditions and Sampling

Grape berry samples were collected in 2018 from a white cultivar (*Vitis vinifera* L. cv. Alvarinho) in the commercial vineyard Quinta Cova da Raposa in the Demarcated Region of Vinho Verde. Vineyard maintenance and sampling methods were as described in Garrido et al. [31]. Two distinct natural light microclimates in the grapevine canopy—without any defoliation treatment—were considered for harvesting: low light (LL) clusters that grew in the shaded inner zones of the canopy (approx. 50 μmol photons m^−2^ s^−1^ on average), and high light (HL) clusters that were exposed to direct sunlight most of the day (approx. 150 μmol photons m^−2^ s^−1^ on average) [31]. At the same time of the day (around 9 a.m.), the grape berries clusters were randomly collected considering all the grapevines of the field trial, as 3 biological replicates, from both light microclimates and at 3 different developmental stages: green (16th July, 6 weeks after anthesis (WAA) or BBCH-75—BBCH-scale used for grapes by Lorenz et al. [81]), *véraison* (29th August, 12 WAA, BBCH-83), and mature (17th September, 15 WAA, BBCH-89). At each developmental stage, the grape berry clusters were first visually analyzed to avoid phenological displacements between them. Each biological replicate corresponds to a mixture of 15 to 20 berries from 3 to 5 clusters from 6 to 8 plants growing in untreated vineyard plots (i.e., from the non-irrigated, non-kaolin controls plants, as described in Garrido et al. [31]). The whole berries were transported from the field to the laboratory in refrigerated boxes (within 10–15 min) and then immediately frozen in liquid nitrogen and stored at −80 °C. Prior to the analysis, for each condition, one or more subsamples of grapes were prepared from each triplicate. The exocarps and seeds were separated and ground to a fine powder using a mortar and pestle under liquid nitrogen and stored until analysis or immediately used. 

### 3.2. RNA Extraction and cDNA Synthesis

RNA was extracted from a total of 36 samples: 3 subsamples, 1 from each biological replicate × 2 tissues × 2 microclimates × 3 developmental stages. Total RNA was purified according to Reid et al. [82], with some adjustments. To 500 mg of frozen tissue, 3 mL of the extraction buffer containing 2% of cetrimonium bromide (CTAB), 2% of soluble polyvinylpyrrolidone (PVP) K-30, 300 mM TRIS-HCl (pH 8.0), 25 mM of ethylenediamine tetraacetic acid (EDTA), 2 M of sodium chloride (NaCl), and 40 mM of dithiothreitol (DTT, mixed just prior to use) were added. Samples were incubated at 60 °C for 30 min and shaken every couple of minutes. After this, the mixtures were extracted twice with 3 mL of chloroform:isoamyl alcohol (24:1) followed by a centrifugation step at 3500× *g* for 15 min at 4 °C. The aqueous fraction (1.5 mL) was mixed with 0.1 vol of 3 M NaOAc (pH 5.2) and 0.6 vol of isopropanol, and maintained at −80 °C for 30 min, after which the samples were centrifuged at 3500× *g* for 30 min at 4 °C. The pellet was resuspended in 500 μL of plant RNA lysis solution from a GeneJET Plant RNA Purification Mini Kit (Thermo Scientific^®^, Waltham, MA, USA), following the manufacturer’s instructions. RNA concentration was determined in a Nanodrop (Thermo Fisher Scientific Inc., Waltham, MA, USA) and its integrity was assessed in a 1% agarose gel. Total RNA was further purified with DNase I Kit (Thermo Scientific^®^, Waltham, MA, USA) to remove any contaminating DNA. First strand cDNA synthesis was synthesized from 1 μg of total RNA using the Xpert cDNA Synthesis Mastermix (Grisp^®^, Porto, Portugal), following the manufacturer’s instructions.

### 3.3. Transcriptional Analysis by Real-Time qPCR

Real-time qPCR was used for transcriptional analyses of target genes listed in the Table S1 (in the Supplementary Material section). The gene specific primer pairs used for each target or reference gene are also listed in Table S1. The primers of the target genes were designed using the software QuantPrime [83]. The analysis was performed with Xpert Fast SYBR (uni) Blue (Grisp^®^) using 1 μL cDNA (diluted 1:10 in ultra-pure distilled water) in a final reaction volume of 10 μL per well. 

Experiments were performed in triplicate, as described above, in an CFX96 Real-Time Detection System (Bio-Rad, Foster City, CA, USA) using the following cycler conditions: polymerase was activated with an initial step of 3 min at 95 °C, the double strand denaturation occurred at 95 °C for 10 s, the annealing temperature was 55 °C for 20 s and the extension temperature was 72 °C for 20 s (amplification was performed using 40 cycles). Melting curve analysis was performed for specific gene amplification confirmation. 

The reference genes actin 1 (*VvACT1*) and glyceraldehyde-3-phosphate dehydrogenase (*VvGAPDH*) were selected, because these genes were proven to be highly stable and ideal for qPCR normalization purposes in grapevine [82]. The primers used for their amplification by qPCR were those described in Reid et al. [82]. Additionally, for each qPCR analysis, the target gene stability (i.e., the absence of significant variation of the expression of the reference genes in all samples), was validated by the M-values and coefficient variance values calculated by CFX ManagerTM Software (Bio-Rad): for these parameters, the acceptable values for the stability should be less than 1 and 0.5, respectively [84]. Then, the expression values were normalized by the average of the expression of the reference genes, as described by Pfaffl [85], and the results shown as arbitrary units (a.u.) of relative expression.

### 3.4. Enzyme Assays

The activity of two enzymes (i.e., flavonol synthase—FLS, EC 1.14.20.6; and anthocyanidin reductase—ANR, EC 1.3.1.77), key to the biosynthesis of flavonols and flavan-3-ols, respectively, was also assessed, as described below, to further analyze the observed changes in the gene expression at the enzyme activity level. For these assays, for each microclimate and developmental stage, grape berry tissues were prepared using 5 (for FLS) and 4 to 8 (for ANR) subsamples from the biological replicates, as described above.

#### 3.4.1. Enzyme Extraction

Total protein extraction from grape berry powders was performed mainly as described by Stoop and Pharr [86]. One gram of sample powder was mixed with 1 mL of buffer containing 50 mM bis-tris propane (pH 8.9, adjusted with HCl), 5 mM MgCl_2_, 1 mM EDTA, 1 mM phenylmethylsulfonyl fluoride (PMSF), 5 mM dithiothreitol (DTT), and 10 mg of polyvinylpolypyrrolidone (PVPP). The homogenates were thoroughly mixed and centrifuged at 18,000× *g* for 20 min and the supernatants were maintained on ice and used for all enzyme assays. Total protein concentrations of the extracts were determined by the Bradford method [87], reading at 595 nm and using bovine serum albumin (Sigma-Aldrich Algés, Portugal) as a standard.

#### 3.4.2. Flavonol Synthase (FLS) Activity

FLS activity was performed as described by Conde et al. [64], with some modifications. The activity was determined spectrophotometrically (Shimadzu UV-1700) following quercetin production at 365 nm, for 30 min at 37 °C, in a total volume of 1 mL. The reaction medium (pH = 5.0) contained 85.77 mM of phosphate-buffered saline (PBS) buffer, 111 mM of sodium acetate, 83 µM of 2-oxoglutaric acid, 131 µM of taxifolin (dihydroquercetin), and 100 µL of protein extract, and started with 84 µM of ferrous sulfate. FLS activity was calculated using the extinction coefficient of quercetin (ε = 13.2 mM^−1^ cm^−1^) and expressed in nmol_quercetin_ min^−1^ mg^−1^ of protein, that is, equivalent to 1 mU per mg of protein (the enzyme unit, U, corresponds to the conversion of one µmol of substrate per minute).

#### 3.4.3. Anthocyanidin Reductase (ANR) Activity

ANR activity was determined following NADPH consumption at 45 °C, in a total reaction volume of 1.5 mL with 89 mM of PBS buffer (pH = 6.5), 133 µM of nicotinamide adenine dinucleotide phosphate (NADPH), 66.7 µM of cyanidin chloride, 533 µM of ascorbic acid, and 60 µL of protein extract. The enzyme activity was monitored spectrophotometrically (Shimadzu UV-1700) by the rate of NADPH oxidation at 340 nm, after adding its substrate cyanidin chloride. ANR activity was calculated using the extinction coefficient of NADPH (ε = 6.22 mM^−1^ cm^−1^) and expressed in nmol_NADPH_ oxidized min^−1^ mg^−1^ protein, that is, equivalent to 1 mU per mg of protein.

### 3.5. Statistical Analysis

Data from gene expression was first transformed (Log(X+1)) to meet homogeneity of variances. Then, a two-way ANOVA was applied followed by the post hoc Bonferroni test for multiple comparisons whenever the factors (microclimate or developmental stage) had a significant effect (GraphPad Prism version 5.00 for Windows, GraphPad Software, La Jolla, CA, USA). Significant differences (*p* ≤ 0.05) between sample groups are indicated with different letters: capital letters refer to differences between developmental stages for the same microclimate, and lowercase letters refer to differences between microclimates for each stage.

A correlation analysis between the relative expression levels of a selected set of genes (i.e., *VvFLS1*, *VvDFR*, *VvLDOX*, *VvLAR1*, *VvLAR2*, *VvANR*, and *VvMYBPA1*) was undertaken. The Pearson correlation coefficients (*r*) were calculated separately for both exocarp and seed, considering all samples regardless of the light microclimate and developmental stage (*n* = 18 per tissue). The calculations of *r* and *p*-values were performed using Microsoft Excel^®^ (version 2008: 13127.21624), and the correlation matrices for each tissue are presented in the Supplementary Material section (Tables S2 and S3).

## 4. Conclusions

In general, the results obtained in this study corroborate those of our previous studies [31,32], suggesting that tissue-specific photosynthesis coincides with the expression of photosynthesis and sucrose synthesis-related genes, and to the gene transcription and enzyme activities of key steps in secondary metabolism. Our results point to a possible cross-talk between photosynthesis and sucrose unloading and breakdown/carbon usage in the berries, and with the secondary metabolism. In the green stage, the berries have a relatively high demand for carbon and energy to sustain their high growth rate, and the main part of the sucrose imported through the dorsal vascular system is used to meet that demand: the relatively high levels of transcripts of genes involved in sucrose catabolism (*VvSuSy1*), combined with the low levels of *VvSPS1* in the exocarp, may support our hypothesis that photosynthesis contributes energy to that unloading process. In addition, the high expression levels of genes from the phenylpropanoid (*VvPAL1*) and stilbenoid (*VvSTS1*) pathways, as well as of those associated with flavan-3-ol biosynthesis (*VvDFR*, *VvLAR2*, and *VvANR*) in the exocarp at the green stage, may indicate a possible cross-talk/relationship between photosynthesis and secondary metabolism, by supplying energy and/or carbon-skeletons for these pathways. In the exocarp, the flavonol branch of the flavonoid pathway (*VvFLS1*) was up-regulated by the HL microclimate compared to the LL microclimate, and increased with ripening. In parallel, the expression of *VvChlSyn*, *VvRuBisCO*, and genes involved in the violaxanthin cycle (*VvVDE1* and *VvZEP1*) increased during ripening; these changes are possibly associated with photoprotection. The results of seeds generally indicate that some genes peaked in their expression at either the *véraison* or mature stages (i.e., the xanthophyll cycles—*VvVDE1* and *VvLUT1*; phenylpropanoids—*VvCHS3*; and the flavan-3-ol pathway—*VvDFR* and *VvLAR2*). Thus, it appears that, at later stages of berry development, additional mechanisms other than the assimilation of atmospheric CO_2_ fixation may be involved to maintain the need for carbons for primary and secondary metabolism in the berries. *VvRuBisCO* expression was maintained during ripening in both berry tissues. At the later stages of berry development, in particular, this RuBisCO may have a function in re-assimilating the locally released CO_2_ (from respiration or from decarboxylative reactions, such as malate catabolism), thus contributing the substrates required for the various metabolic pathways that are active in these berry tissues.

Clearly, further molecular and biochemical studies are needed to support the proposed link between the actual photosynthesis in these grape berry tissues and the physiological/biochemical/transcriptional changes observed in these tissues [31,32]. These fundamental scientific studies will also contribute essential information to the viticulture practices that involve manipulations of the canopy light microclimate.

## Data Availability

Not applicable.

## Supplementary Materials

The following are available online at https://www.mdpi.com/article/10.3390/plants10091769/s1, Table S1: Forward (F) and reverse (R) primers used for gene expression analysis by real-time PCR. Figure S1: Simplified carotenoid metabolic pathway. Figure S2: Biosynthetic pathways of phenolic compounds in grape berry [88,89]. Table S2: Matrix with Pearson correlation coefficients (r), calculated for selected genes in the exocarp. Table S3: Matrix with Pearson correlation coefficients (r), calculated for selected genes in the seed.
