# Supplementary material for: Light Microclimate-Driven Changes at Transcriptional Level in Photosynthetic Grape Berry Tissues
Source: Plants (Basel). 2021 Aug 25;10(9):1769. doi: 10.3390/plants10091769 (PMC8465639; doi:10.3390/plants10091769)
Supplement: Supplementary file 1 [file plants-10-01769-s001.zip › plants-1294616-supplementary.pdf]

Light Microclimate-Driven Changes at Transcriptional Level in Photosynthetic Grape Berry Tissues

Andreia Garrido [1,2,*], Ric C. H. De Vos [3], Artur Conde [1,2,†] and Ana Cunha [1,2,4,*,†]

[1] Centre of Molecular and Environmental Biology (CBMA), Department of Biology, University of Minho, Campus de Gualtar, 4710-057 Braga, Portugal; arturconde@bio.uminho.pt
[2] Centre for the Research and Technology of Agro-Environmental and Biological Sciences (CITAB), University of Trás-os-Montes e Alto Douro, Quinta de Prados, 5000-801 Vila Real, Portugal
[3] Business Unit Bioscience, Wageningen Plant Research, Wageningen University and Research (Wageningen-UR), PO Box 16, 6700 AA Wageningen, The Netherlands; ric.devos@wur.nl
[4] Centre of Biological Engineering (CEB), University of Minho, Campus de Gualtar, 4710-057 Braga, Portugal
* Correspondence: andreiagarrido@sapo.pt (A.G.); accunha@bio.uminho.pt (A.C.)
† Equal senior authorship.

Supplementary Materials

Table S1. Forward (F) and reverse (R) primers used for gene expression analysis by real-time PCR. Sequences accession numbers were obtained through Grape Genome Browser 12x or, for the carotenoid metabolism genes, from Plaza 1.0 (April, 2009; http://bioinformatics.psb.ugent.be/plaza_v1/), or Grape Genome Database (http://genomes.cribi.unipd.it/grape/) for *VvLAR2* and *VvRubisCO*.

Gene	Accession number (Genoscope)	Primers
VvACT1 (reference gene)	GSVIVT01026580001	F: 5′-GTGCCTGCCATGTATGTTGCC-3′
		R: 5′-GCAAGGTCAAGACGAAGGATA-3′
VvGAPDH (reference gene)	GSVIVT00009717001	F: 5′-CACGGTCAGTGGAAGCATCAT-3′
		R: 5′-CCTTGTCAGTGAACACACCAG-3′
VvPAL1	GSVIVG01025703001	F: 5′-CCGAACCGAATCAAGGACTG-3′
		R: 5′-GTTCCAGCCACTGAGACAAT-3′
VvSTS1	GSVIVT01010590001	F: 5′-CGAAGCAACTAGGCATGTGT-3′
		R: 5′-CTCCCCAATCCAATCCTTCA-3′
VvCHS1	GSVIVT01032968001	F: 5′-GTCCCAGGGTTGATTTCCAA-3′
		R: 5′-TCTCTTCCTTCAGACCCAGTT-3′
VvCHS3	Lac14-SSH (CX126991)	F: 5′-TCGCATCACAAATAGCGAAC-3′
		R: 5′-CAGGGAAGCTGCCATGTATT-3′
VvFLS1	GSVIVT01008913001	F: 5′-CAGGGCTTGCAGGTTTTTAG-3′
		R: 5′-GGGTCTTCTCCTTGTTCACG-3′
VvMYBPA1	GSVIVT01010590001	F: 5′-AGATCAACTGGTTATGCTTGCT-3′
		R: 5′-AACACAAATGTACATCGCACAC-3′
VvDFR	GSVIVT01009743001	F: 5′-GGCTTTCTAGCGAGAGCGTA-3′
		R: 5′-ACTCTCATTTCCGGCACATT-3′
VvANR	GSVIVG01035256001	F: 5′-CAATACCAGTGTTCCTGAGC-3′
		R: 5′-AAACTGAACCCCTCTTTCAC-3′
VvLAR1	GSVIVT01024419001	F: 5′-CAGGAGGCTATGGAGAAGATAC-3′
		R: 5′-ACGCTTCTCTCTGTACATGTTG-3′
VvLAR2	VIT_217s0000g04150	F: 5′-CAAATCGCTTCATTTCCGACCTCCC-3′
		R: 5′-GTCATCTTCTTCCACGGTTACACGG-3′
VvLDOX	GSVIVT01009743001	F: 5′-ACCTTCATCCTCCACAACAT-3′
		R: 5′-AGTAGAGCCTCCTGGGTCTT-3′
VvChlSyn	GSVIVT01011187001	F: 5′-GCTGAGACTGATGCAAATGAAGCC-3′
		R: 5′-TGCCGAGAAGCTGATTGAAGTTCG-3′
VvRubisCO	VIT_13s0019g02050	F: 5′-TTCAAAGCTCTGCGCGCTCTAC3′
		R: 5′-TCTCTCAACTTGGATGCCATGAGG-3′
VvSPS1	GSVIVT01012825001	F: 5′-GGTTTGCCTATTGTTGCCACCAG-3′
		R: 5′-CAAGAAGGCCATTGTCAAGTACCC-3′
VvSuSy1	GSVIVT01015018001	F: 5′-TGTTAAGGCTCCTGGATTTCAATTA-3′
		R: 5′-AGCCAAATCTTGGCAAGCA-3′
VvPSY1	VV00G37410	F: 5′-TCGATGTGGTGAAGTTTGTGCAG-3′
		R: 5′-TCAGGTGTCATCAGCATTGTTCCC-3′
VvLBCY2	VV08G15130	F: 5′-AGGCTGGGTGGTTGAGAATTGG-3′
		R: 5′-TCCTCAACGACAAACCGAAGTGG-3′
VvLECY1	VV11G01840	F: 5′-TGGATGTGGCAGGGATTTCTTGG-3′
		R: 5′-TAGCATGGTTGCTCCAGTAGGG-3′

VvLUT1	VV08G10880	F: 5′-TGCTTGCTAGCAGAGAAGAGGTG-3′
		R: 5′-ACTGCCATTGAGTGCATCAGTTC-3′
VvVDE1	VV00G14320	F: 5′-GTTGTCTATGGTTGGTGGTTTGGC-3′
		R: 5′-ATGCCAGTGGGTCTCCATTTCC-3′
VvZEP1	VV07G11310	F: 5′-AGCGTGCTATTGGTGGAGAGTG-3′
		R: 5′-CCCGATTATGCAGGGTTTGTTCTC-3′

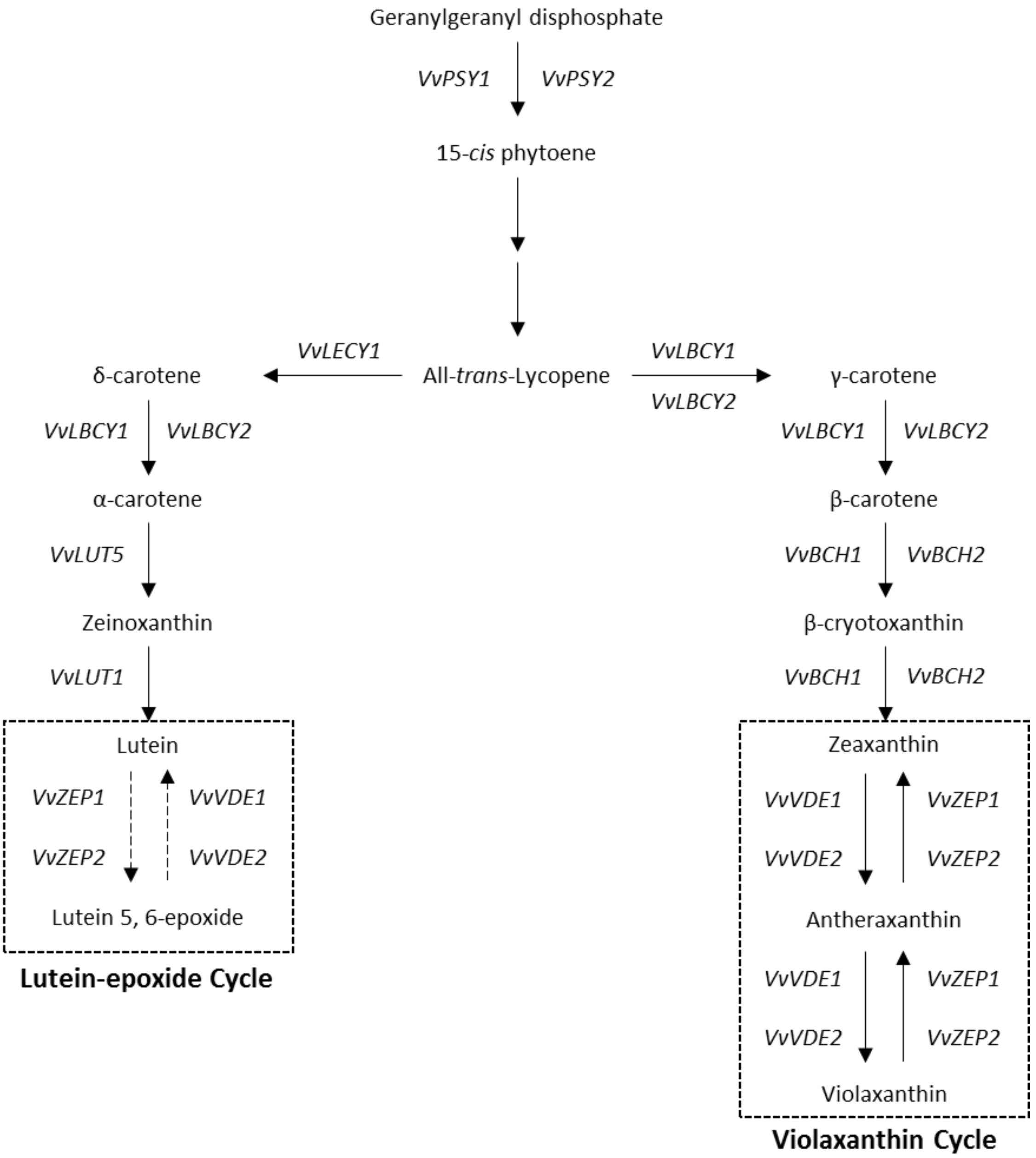

Figure S1. Simplified carotenoid metabolic pathway. *PSY1*/*PSY2*, phytoene synthase; *LECY1*/*LECY2*, lycopene epsilon cyclase; *LBCY1*/*LBCY2*, lycopene β-cyclase; *LUT5*/*LUT1*, lutein synthase; *BCH1*/*BCH2*, β-carotene hydroxylase; *VDE1*/*VDE2*, violaxanthin de-epoxidase; *ZEP1*/*ZEP2*, zeaxanthin epoxidase. Adapted from Young et al. [39].

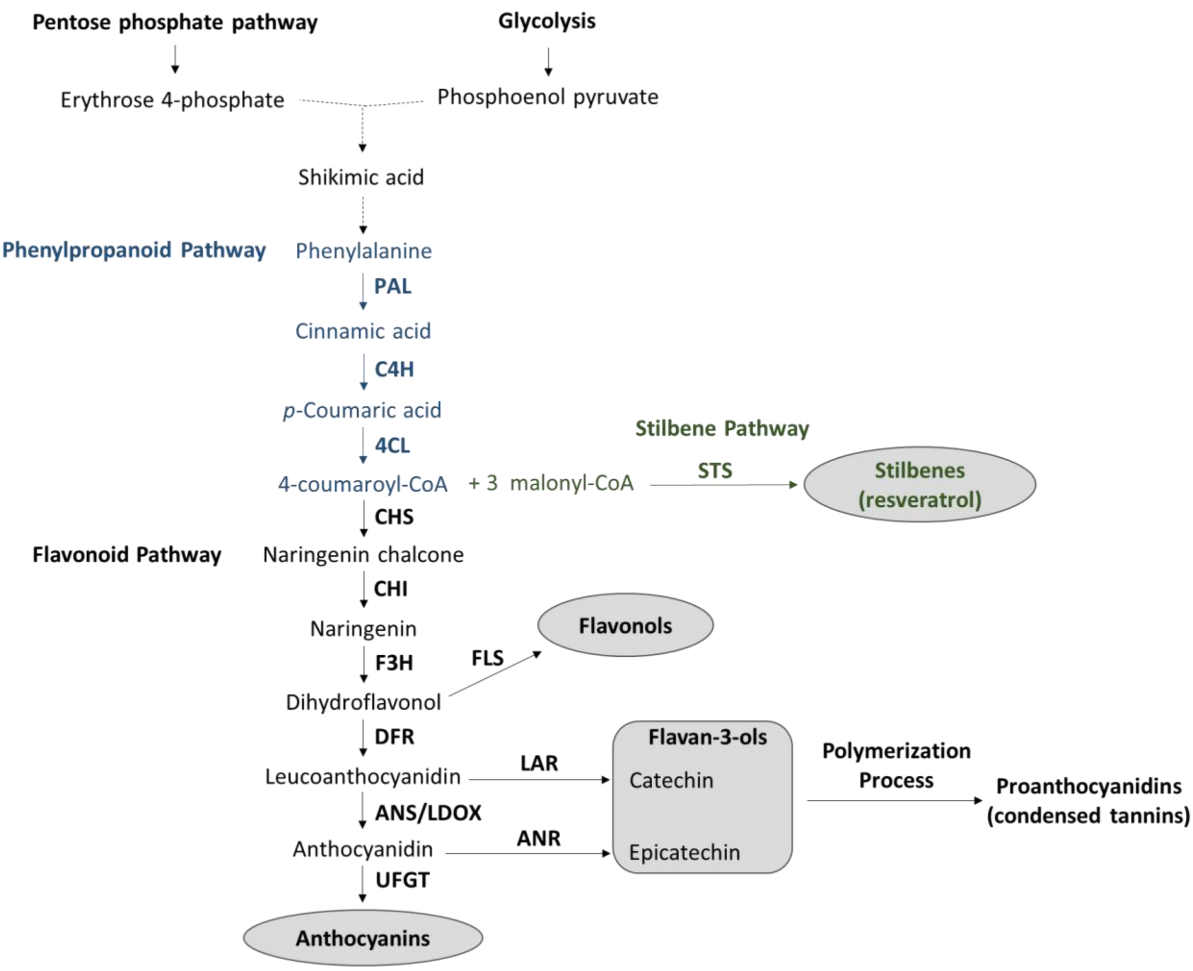

Figure S2. Biosynthetic pathways of phenolic compounds in grape berry. PAL, phenylalanine ammonia lyase; C4H, cinnamate-4-hydroxylase; 4CL, 4-coumaroyl:CoA-ligase; CHS, chalcone synthase; STS, stilbene synthase; CHI, chalcone isomerase; F3H, flavonone 3-hydroxylase; FLS, flavonol synthase; DFR, dihydroflavonol reductase; ANS, anthocyanidin synthase; LDOX, leucoanthocyanidin dioxygenase; LAR, leucoanthocyanidin reductase; ANR, anthocyanidin reductase; UFGT, flavonoid glucosyltransferase. Full lines represent direct enzymatic conversion and dashed lines represent omitted intermediates. Adapted from Ageorges et al. [88] and Teixeira et al. [89].

Table S2. Matrix with Pearson correlation coefficients (r), calculated for selected genes (*VvFLS1*, *VvDFR*, *VvLDOX*, *VvLAR1*, *VvLAR2*, *VvANR* and *VvMYBPA1*) in the exocarp, considering all samples collected in both light microclimate and developmental stages (n = 18). The coefficient values in bold are statistically significant ($p \leq 0.05$).

	VvFLS1	*VvDFR*	*VvLDOX*	*VvLAR1*	*VvLAR2*	*VvANR*	*VvMYBPA1*
VvFLS1	1.000						
VvDFR	**-0.770**	1.000					
VvLDOX	-0.419	-0.104	1.000				
VvLAR1	-0.439	0.456	-0.021	1.000			
VvLAR2	-0.452	**0.842**	-0.414	**0.567**	1.000		
VvANR	**-0.504**	**0.845**	-0.381	**0.567**	**0.982**	1.000	
VvMYBPA1	**-0.519**	**0.864**	-0.335	**0.557**	**0.962**	**0.979**	1.000

Table S3. Matrix with Pearson correlation coefficients (*r*), calculated for selected genes (*VvFLS1*, *VvDFR*, *VvLDOX*, *VvLAR1*, *VvLAR2*, *VvANR* and *VvMYBPA1*) in the seed, considering all samples collected in both light microclimate and developmental stages (n = 18). The coefficient values in bold are statistically significant ($p \leq 0.05$).

	VvFLS1	*VvDFR*	*VvLDOX*	*VvLAR1*	*VvLAR2*	*VvANR*	*VvMYBPA1*
VvFLS1	1.000						
VvDFR	-0.379	1.000					
VvLDOX	**0.877**	-0.399	1.000				
VvLAR1	-0.239	0.367	-0.342	1.000			
VvLAR2	-0.167	**0.752**	-0.138	0.327	1.000		
VvANR	**0.556**	0.098	0.357	0.420	0.212	1.000	
VvMYBPA1	0.125	**0.698**	0.167	0.215	**0.782**	0.404	1.000
